# Diagnosis of architectural distortion on digital breast tomosynthesis using radiomics and deep learning

**DOI:** 10.3389/fonc.2022.991892

**Published:** 2022-12-13

**Authors:** Xiao Chen, Yang Zhang, Jiahuan Zhou, Xiao Wang, Xinmiao Liu, Ke Nie, Xiaomin Lin, Wenwen He, Min-Ying Su, Guoquan Cao, Meihao Wang

**Affiliations:** ^1^ Department of Radiology, Key Laboratory of Intelligent Medical Imaging of Wenzhou, First Affiliated Hospital of Wenzhou Medical University, Wenzhou, China; ^2^ Department of Radiation Oncology, Rutgers-Cancer Institute of New Jersey, Robert Wood Johnson Medical School, New Brunswick, NJ, United States; ^3^ Department of Radiological Sciences, University of California, Irvine, Irvine, CA, United States; ^4^ Department of Radiology, Yuyao Hospital of Traditional Chinese Medicine, Ningbo, China; ^5^ School of Laboratory Medicine and Life Sciences, Wenzhou Medical University, Wenzhou, China; ^6^ Department of Medical Imaging and Radiological Sciences, Kaohsiung Medical University, Kaohsiung, Taiwan

**Keywords:** architectural distortion, breast cancer diagnosis, deep learning, digital breast tomosynthesis, radiomics

## Abstract

**Purpose:**

To implement two Artificial Intelligence (AI) methods, radiomics and deep learning, to build diagnostic models for patients presenting with architectural distortion on Digital Breast Tomosynthesis (DBT) images.

**Materials and Methods:**

A total of 298 patients were identified from a retrospective review, and all of them had confirmed pathological diagnoses, 175 malignant and 123 benign. The BI-RADS scores of DBT were obtained from the radiology reports, classified into 2, 3, 4A, 4B, 4C, and 5. The architectural distortion areas on craniocaudal (CC) and mediolateral oblique (MLO) views were manually outlined as the region of interest (ROI) for the radiomics analysis. Features were extracted using PyRadiomics, and then the support vector machine (SVM) was applied to select important features and build the classification model. Deep learning was performed using the ResNet50 algorithm, with the binary output of malignancy and benignity. The Gradient-weighted Class Activation Mapping (Grad-CAM) method was utilized to localize the suspicious areas. The predicted malignancy probability was used to construct the ROC curves, compared by the DeLong test. The binary diagnosis was made using the threshold of ≥ 0.5 as malignant.

**Results:**

The majority of malignant lesions had BI-RADS scores of 4B, 4C, and 5 (148/175 = 84.6%). In the benign group, a substantial number of patients also had high BI-RADS ≥ 4B (56/123 = 45.5%), and the majority had BI-RADS ≥ 4A (102/123 = 82.9%). The radiomics model built using the combined CC+MLO features yielded an area under curve (AUC) of 0.82, the sensitivity of 0.78, specificity of 0.68, and accuracy of 0.74. If only features from CC were used, the AUC was 0.77, and if only features from MLO were used, the AUC was 0.72. The deep-learning model yielded an AUC of 0.61, significantly lower than all radiomics models (p<0.01), which was presumably due to the use of the entire image as input. The Grad-CAM could localize the architectural distortion areas.

**Conclusion:**

The radiomics model can achieve a satisfactory diagnostic accuracy, and the high specificity in the benign group can be used to avoid unnecessary biopsies. Deep learning can be used to localize the architectural distortion areas, which may provide an automatic method for ROI delineation to facilitate the development of a fully-automatic computer-aided diagnosis system using combined AI strategies.

## Introduction

Breast cancer is the most prevalent among all cancers in the world (1). In 2020, there were 2.3 million women diagnosed with breast cancer with 685,000 deaths globally. The average risk of a woman in the United States developing breast cancer during her lifetime is about 13%; that is, 1 in 8 women will be diagnosed (2). In China, breast cancer is the most common and rapidly increasing female malignancy (3). Compared with developed countries, the prognosis is much poorer, which varies in different geographic regions (4). The 5-year survival rates during 2003-2015 are from 73.1% to 82.0% (55.9% to 72.9% for rural women), lower than that of 90% for American women (5). With improved health care, the death rate decreased by 1% per year from 2013 to 2018 (6). These decreases are thought to be the results of better treatments, and earlier detection through screening using mammography and ultrasound (2, 6–8).

Early signs of breast cancer on mammography include microcalcifications, mass (space-occupying density), architectural distortion, and bilateral asymmetry (9, 10). Microcalcifications and masses have been studied extensively. Architectural distortion is the third most suspicious appearance, representing 6% of abnormalities detected on screening mammography (11). In the Breast Imaging Reporting and Data System (BI-RADS) lexicon (12), architectural distortion is defined as “the normal architecture of the breast is distorted with no definite mass visible”. This includes spiculations radiating from a point and focal retraction or distortion at the edge of the parenchyma. However, the detection and interpretation of architectural distortion on 2-dimensional (2D) mammograms is challenging. Due to the overlapping tissues, the appearance may be subtle, and it is subjective for radiologists to detect these abnormalities, especially when there are co-existence of other findings such as mass and asymmetry (13).

Since the approval of the digital breast tomosynthesis (DBT) by the U.S. Food and Drug Administration (FDA) in 2011, it has become a widely used imaging modality for screening and diagnosis (14). DBT generates multiple images using scans taken from different angles, and thus can better resolve overlapping tissues. Some countries have recommended either digital mammography (DM) or DBT as appropriate for screening (15–17). Compared with DM, DBT can provide a better morphological characterization of invasive cancers, while mitigating false-positive diagnosis from the superposition of normal parenchyma (18–20). The high sensitivity of DBT for architectural distortion allows for improved diagnosis of invasive ductal cancers (21–24), but many benign diseases will be detected as suspicious, and lead to unnecessary biopsies. DBT may also provide better visualization for invasive lobular cancers, which were difficult to be detected on DM (14).

Recently, artificial intelligence (AI) algorithms have been extensively applied in the medical field. Radiomics with machine learning, and deep learning using convolutional neural network (CNN), have been applied to analyze images for detection and diagnosis of lesions in various clinical applications (25–27). Several studies have applied AI for the detection of architectural distortion (13, 28, 29). Rehman et al. proposed an automated computer-aided diagnostic system using computer vision and deep learning to predict breast cancer based on the architectural distortion on DM (13). Bahl et al. performed a retrospective review and concluded that the presence of architectural distortion on mammography indicated malignancy in approximately 75% of cases (30). In another study, Shu et al. proposed a region-based pooling architecture using a deep convolutional neural network to classify mammography images (31). Most studies reported so far were performed using 2D mammography. Since DBT can provide better spatial information for detection and characterization of architectural distortion, AI can be applied to develop fully-automatic computer-aided diagnostic systems (32, 33).

The purpose of this study is to implement radiomics and deep learning to build diagnostic models for patients presenting with architectural distortion on DBT images. The radiomic analysis was based on the manually outlined region of interest (ROI) by radiologists for extracting features associated with the architectural distortion. Then, the Support Vector Machine (SVM) algorithm was implemented to evaluate the feature importance, and select features to build the classification model to differentiate benign vs. malignant lesions. The deep-learning method was performed using the entire image as input, without any pre-selection to only include the abnormal regions. The algorithm will be self-trained to diagnose breast cancer, that is, to predict that there is a malignant lesion somewhere in the DBT image. The Gradient-weighted Class Activation Mapping (Grad-CAM) method was utilized to localize the suspicious areas that were focused on, including architectural distortions, so it may provide a potential method for automatic ROI delineation. Potentially, the suspicious area detected by deep learning can be combined with radiomics to generate an automatic diagnostic tool for architecture distortion.

## Materials and methods

### Datasets

This retrospective study was performed in accordance with the principles of the Helsinki Declaration and was approved by the institutional ethics committee. The need for obtaining written informed consent from the patients was waived. The dataset was identified by reviewing all patients receiving DBT in The First Affiliated Hospital of Wenzhou Medical University from October 2016 to December 2019. The inclusion criteria were: (1) patients presenting with the architectural distortion as the main suspicious finding on DBT; (2) patients receiving biopsy or surgery to obtain tissues for pathological examination. The exclusion criteria were: (1) patients receiving any prior treatment in the breast; (2) no pathologically confirmed diagnosis; (3) poor image quality. Finally, a total of 298 patients were included in this study. The age range was from 21 to 79 years old, with an average of 50.6 years old. The BI-RADS scores of DBT were obtained from the radiology reports, classified into 2, 3, 4A, 4B, 4C, and 5.

### DBT protocol

The standard mode of Amulet Innovality Digital Breast Tomosynthesis System (Fuji Film, Japan), namely small-angle DBT-ST mode, was used to take images. DBT images were taken first, followed by Full-field Digital Mammography (FFDM) images. The DBT angular range of the X-ray tube was ±7.5°, every 1.0° for a total of 15 acquisitions, using the W-Al anode-filter. For FFDM, the W-Rh anode-filter was used. The images were acquired with the standard craniocaudal (CC) and mediolateral oblique (MLO) projections under breast compression.

### Radiomics feature extraction

**Figure 1:**
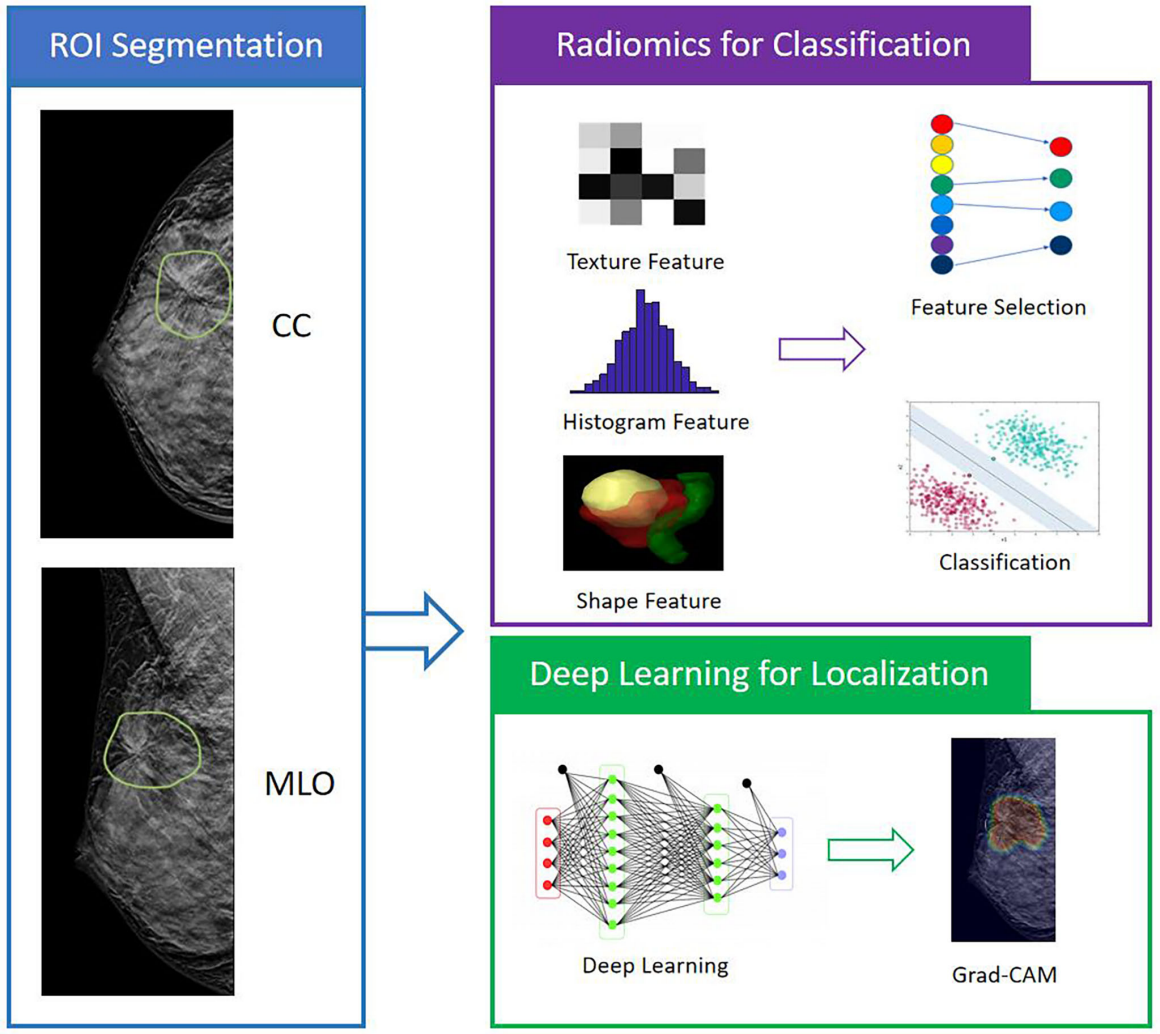
The analysis flowchart. The ROI is manually outlined on the CC and MLO view of one DBT image that shows the most obvious architectural distortion. The radiomic features are extracted using PyRadiomics, and then SVM is applied to select important features and build the classification model to differentiate benign and malignant cases. For the deep learning analysis, the whole image is used as input into ResNet50 to train the diagnostic model. The Gradient-weighted Class Activation Mapping (Grad-CAM) reveals the suspicious area that is focused on to perform classification.

The analysis flowchart is shown in **Figure 1**. The region showing the architectural distortion was delineated by two radiologists based on the consensus through discussion and cross-check. For each patient, only one image that showed the most obvious architectural distortion was used. The region of interest (ROI) was manually drawn using the ImageJ software (https://imagej.nih.gov/ij/index.html). The ROI on CC and MLO images were separately outlined by two junior radiologists first and then examined by a senior radiologist with 7 years of experience interpreting DBT images. If needed, further modification was made. The ROI was resampled into 0.4 × 0.4 mm^2^, and quantized to 25 gray levels. The analysis was performed using PyRadiomics v3.0.1, to extract 107 features including 14 shape, 18 first-order, 24 gray-level co-occurrence matrix (GLCM), 14 gray-level dependence matrix (GLDM), 16 gray-level run length matrix (GLRLM), 16 gray-level size zone matrix (GLSZM), and 5 neighboring gray tone difference matrix (NGTDM) features. For each case, a total of 214 parameters were obtained from the ROI’s drawn on CC and MLO images.

### Feature selection and model building

The feature selection was performed using a sequential method, by constructing multiple SVM classifiers. In this process, SVM with Gaussian kernel was used as the objective function to test the performance of a subset of features using 5-fold cross-validation. In the beginning, an empty candidate set was presented, and features were sequentially added. In each iteration, the training process was repeated 5,000 times to explore the robustness of each feature. After each iteration, the feature that led to the best performance was added to the candidate set. The process stopped when the addition of features no longer met the criterion, i.e., 10^−6^ as the termination tolerance for the objective function value. The algorithm was designed to explore all possible subsets of the ‘‘shadow” attributes and select the final key features by comparing their relative importance. During the feature selection, different class weights were assigned to the benign group and the malignant group to handle the imbalance issue.

After the final features were determined, SVM was used to build the diagnostic model. The performance was evaluated using 10-fold cross-validation, i.e., using 90% cases for training and the remaining 10% for testing. The process was repeated 10 times, and each case could only be included in the testing group once. The radiomics score, i.e. the malignancy probability, was calculated by the model, which was then used for constructing the Receiver Operating Curve (ROC) curve, and making the binary diagnosis using the threshold of ≥ 0.5 as malignant.

### Deep learning analysis

Besides radiomics, deep learning was applied to differentiate the benign and malignant lesions as well as to localize the activation region. The whole image was used as the input. Deep learning was performed using the ResNet50, with the binary output of malignancy and benignity. The input network included the slice along with its two adjacent neighboring slices from CC and MLO. Therefore, the number of input channel was 6. The image was re-sampled to a 256 × 256 matrix using linear interpolation, and then the pixel intensities were normalized to have a mean of 0 and a standard deviation of 1. In contrast to other CNNs, such as VGG or AlexNet that learns features using large convolutional network architectures, the ResNet extracts residual features as subtraction of features learned from the input of that layer using “skip connections”. The ResNet50 architecture contained one 3 × 3 convolutional layer, one max-pooling layer, and 16 residual blocks. Each block contained one 1 × 1 convolutional layer, one 3 × 3 convolutional layer, and one 1 × 1 convolutional layer. The residual connection was from the beginning of the block to the end of the block. The output of the last block was connected to a fully connected layer with a sigmoid function to make the prediction, by providing a malignancy probability. One additional convolutional layer was added to the ResNet50 at the input to reduce the input channel number from 6 to 3.

The dataset was augmented 20 times using random affine transformations, including translation, scaling, and rotation. To avoid overfitting, L2 regularization term was added to the final loss function, and then, during the training process, the early stop was applied based on the lowest validation loss to obtain the optimized model. The loss function was cross-entropy. The training was implemented using the Adaptive Moment Estimation (Adam) optimizer. The learning rate was set to 0.0001 with momentum term β as 0.5 to stabilize training. Parameters were initialized using ImageNet. The batch size was set to 32 and the number of epochs was set to 100. The evaluation was performed using 5-fold cross-validation, 4-fold for training, and 1-fold set aside for testing. Each case had one chance to be included in the testing dataset. The output was a malignancy probability for each case.

In addition to the classification of benign vs. malignant, one great feature of deep learning is the Gradient-weighted Class Activation Mapping (Grad-CAM), which uses the gradient information flowing into the last convolutional layer of the CNN to assign the importance values to each neuron for a particular decision of interest. After the training of ResNet50, DBT images were input into the system. Then the weight maps from the last convolutional layer were extracted. To match the original image size, the extracted maps were interpolated and normalized to a range of [0, 1]. Then these heat maps were overlaid on the original DBT images. To further evaluate the detection of architectural distortions on DBT vs. mammography, the trained model and Grad-CAM from DBT were applied to analyze the corresponding mammography of the same patients.

### Statistical analysis

The U-tests and chi-square tests were used to compare the age and the proportions of BI-RADS between benign and malignant groups, by using SPSS software (version 20.0). The ROC curves generated by the radiomics models built using the CC view, the MLO view, and the combined CC+MLO views were compared using the DeLong test. For each case, the radiomics score was used to make the binary diagnosis of malignant (≥ 0.5) or benign (<0.5). For deep learning, the predicted malignancy probability by the model was used for constructing the ROC curve and making the binary diagnosis. The sensitivity, specificity, and overall accuracy were calculated and compared.

## Results

### Patients’ characteristics and BI-RADS scores

**Figure 2:**
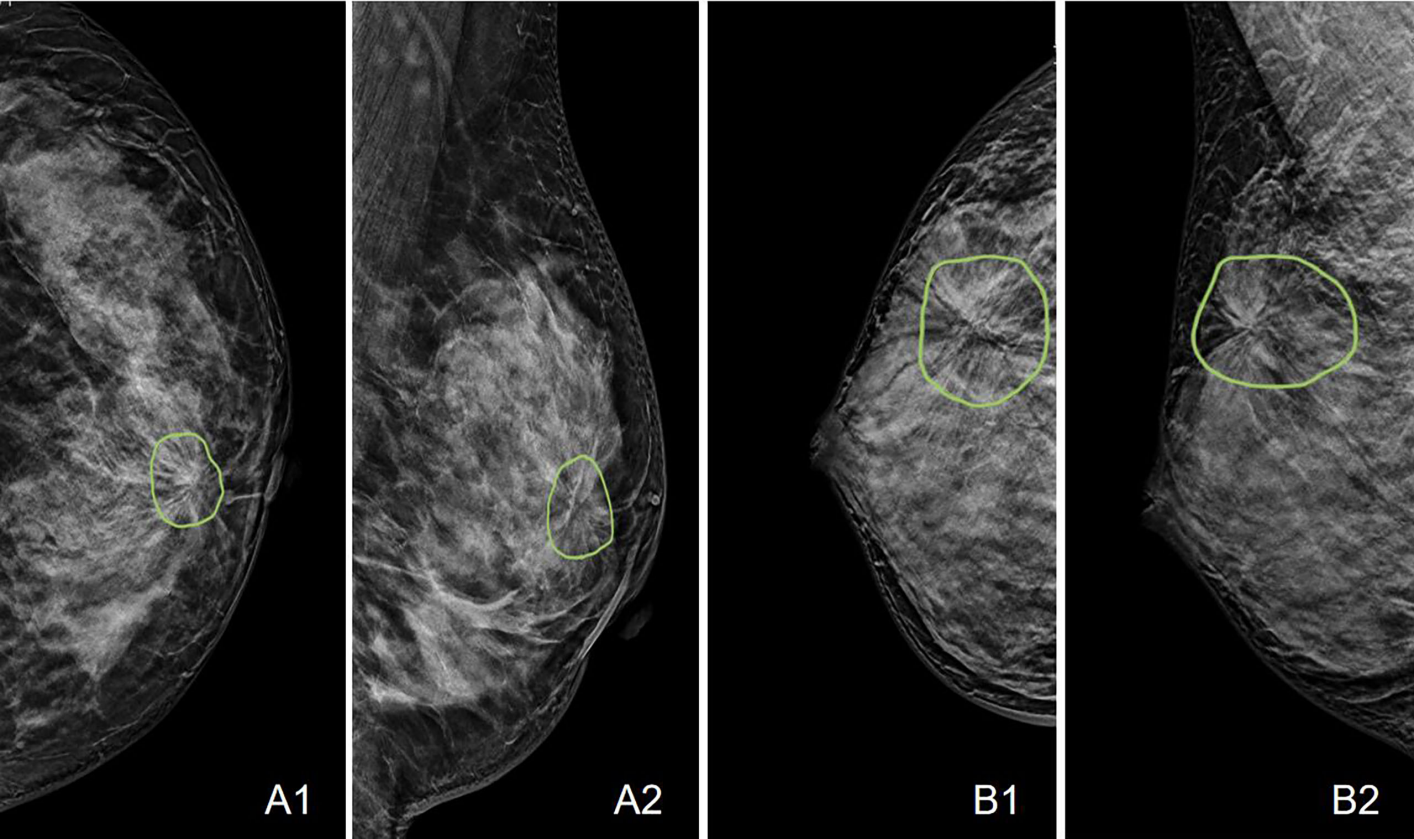
Case examples showing the typical architectural distortion. **A**: The LCC and LMLO views of a 53-year-old patient diagnosed with invasive ductal cancer. The BI-RADS score is 4B. The radiomics score of the combined model is 0.65, correctly diagnosing this case as malignant, true-positive. **B**: The RCC and RMLO views of a 42-year-old patient diagnosed with sclerosing adenosis. The BI-RADS score is 4C. The radiomics score of the combined model is 0.48, correctly diagnosing this case as benign, true-negative.

**Figure 3:**
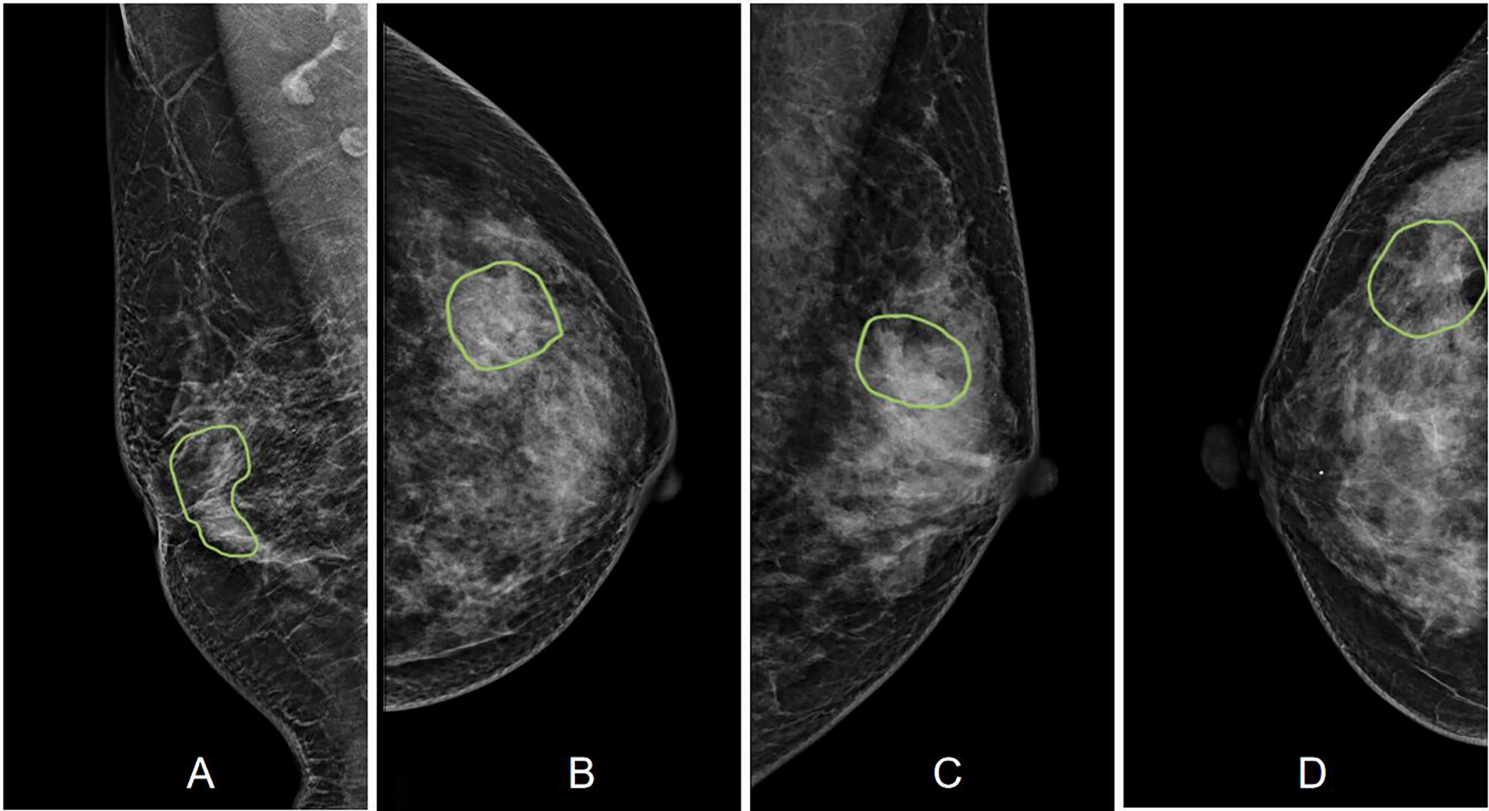
Case examples showing the atypical architectural distortion. **(A)** The RMLO view of a 57-year-old patient diagnosed with invasive ductal cancer. The BI-RADS score is 5. The radiomics score of the combined model is 0.66, true-positive. **(B)** The LCC view of a 39-year-old patient diagnosed with ductal carcinoma *in situ*. The BI-RADS score is 5. The radiomics score of the combined model is 0.61, true-positive. **(C)** The LMLO view of a 39-year-old patient diagnosed with papilloma. The BI-RADS score is 4B. The radiomics score of the combined model is 0.48, true-negative. **(D)** The RCC view of a 52-year-old patient diagnosed with adenosis. The BI-RADS score is 3. The radiomics score of the combined model is 0.41, true-negative.

A total of 175 (59%) malignant and 123 (41%) benign cases were identified. The age and distribution of BI-RADS scores are listed in **Table 1**. The mean age was 52.3 ± 8.7 in the malignant group, and 48.2 ± 8.9 in the benign group. The majority of malignant lesions had BI-RADS scores of 4B, 4C, and 5 (148/175 = 84.6%). In the benign group, a substantial number of patients also had high BI-RADS ≥ 4B (56/123 = 45.5%), but significantly lower than in the malignant groups (p < 0.001). If including 4A, (102/123 = 82.9%) had BI-RADS ≥ 4A, and these patients would be recommended for biopsy and led to the false-positive diagnosis. In the present study, all benign lesions had histological confirmation. The pathological types are listed in **Table 2**. Lobular carcinoma *in situ* (LCIS) is a high-risk pathology and is classified into the malignant group. **Figure 2** shows 2 cases presenting the typical features, and **Figure 3** shows 4 cases presenting the atypical features of architectural distortion. The ROI was drawn to cover the entire area noted as suspicious.

**Table 1:** Age and BI-RADS of lesions determined on DBT in the study cohort.

	Malignant (N=175)	Benign (N=123)	P-Value
**Age [Range]**	52.3 ± 8.7 [29, 79]	48.2 ± 8.9 [21, 73]	0.52
**BI-RADS Score**			0.09
BI-RADS 2	1 (0.6%)	9 (7.3%)	
BI-RADS 3	4 (2.3%)	12 (9.8%)	
BI-RADS 4A	22 (12.6%)	46 (37.4%)	
BI-RADS 4B	54 (30.9%)	46 (37.4%)	
BI-RADS 4C	61 (34.9%)	10 (8.1%)	
BI-RADS 5	33 (18.9%)	0	

**Table 2:** Pathological types of lesions in the study cohort.

	Groups	Case Number (%)
**Pathological Types**	**Malignant**	**Total *N* = 175**
	Invasive Ductal Cancer^a^	133 (76.0%)
	Ductal Carcinoma *In Situ* ^b^	27 (15.4%)
	Invasive Lobular Carcinoma	9 (5.1%)
	Tubular Carcinoma	2 (1.1%)
	Lobular Carcinoma *In Situ*	2 (1.1%)
	Other Cancer	2 (1.1%)
	**Benign**	**Total *N* = 123**
	Adenosis	63 (51.2%)
	Fibroadenoma	42 (34.1%)
	Papilloma	17 (13.8%)
	Other Benign Tumor	1 (0.8%)

a: Main pathology is IDC, may have presence of DCIS or invasive lobular cancer.

b: Main pathology is DCIS, may contain micro invasion of IDC.

### Radiomics analysis

A total of 8 radiomics features were selected to build the final CC+MLO model, in the order of importance: (1) GLCM Cluster Prominence from MLO, (2) NGTDM Coarseness from CC, (3) GLCM Difference Entropy from CC, (4) Skewness from MLO, (5) GLCM Maximum Probability from CC, (6) GLRLM Long Run Emphasis from CC, (7) Interquartile Range from CC, (8) GLDM Dependence Entropy from CC. Among these, 6 were from CC and 2 were from MLO.

**Figure 4:**
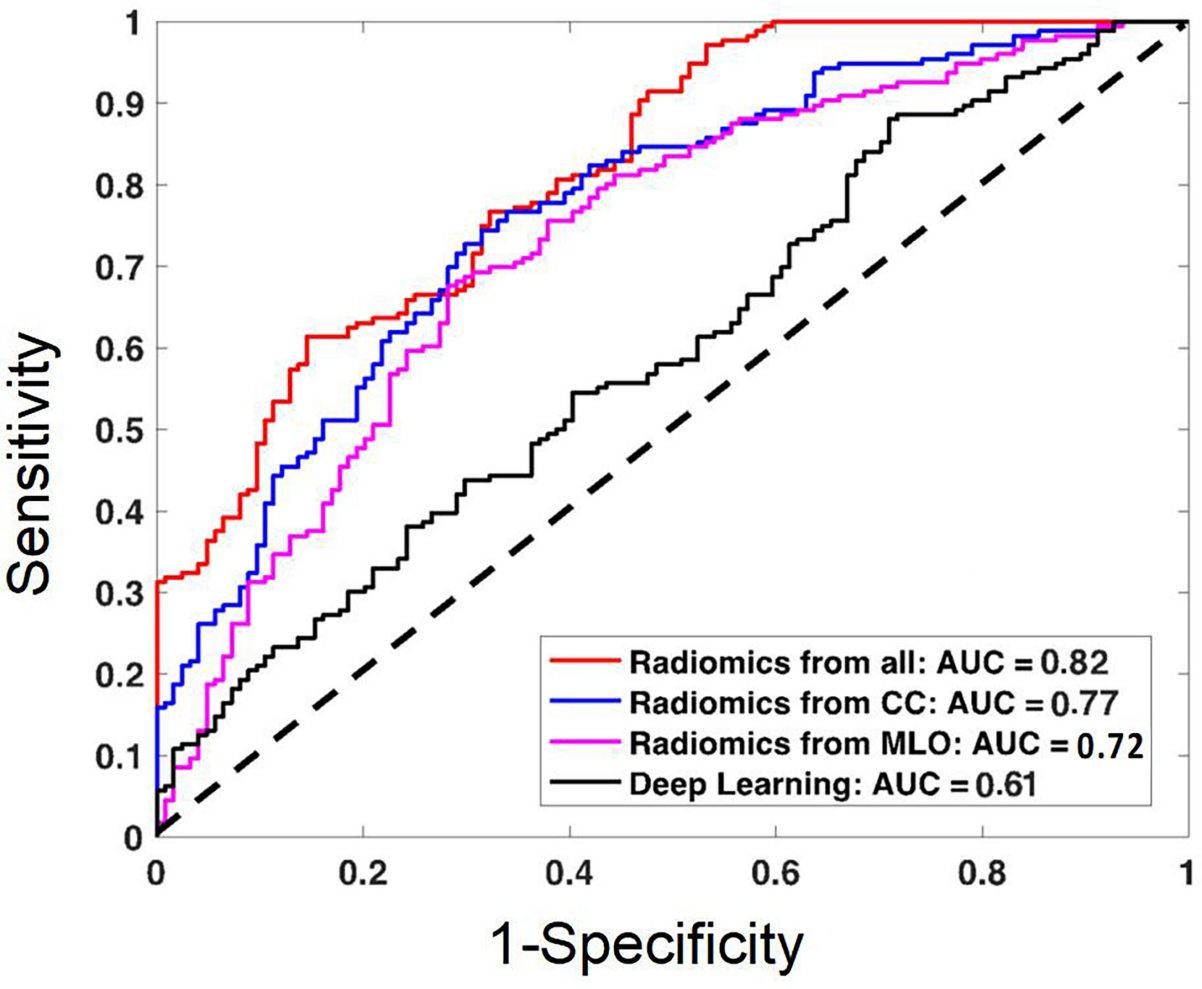
The ROC curves constructed by using the radiomics scores obtained from the models built using the combined CC and MLO features, CC features only, MLO features only; and the ROC curve constructed by using the probability obtained from the deep learning model. The AUC of the combined radiomics model is the highest, 0.82. The AUC of the deep learning model is the lowest, 0.61, likely due to the use of the whole image as the input.

**Figure 5:**
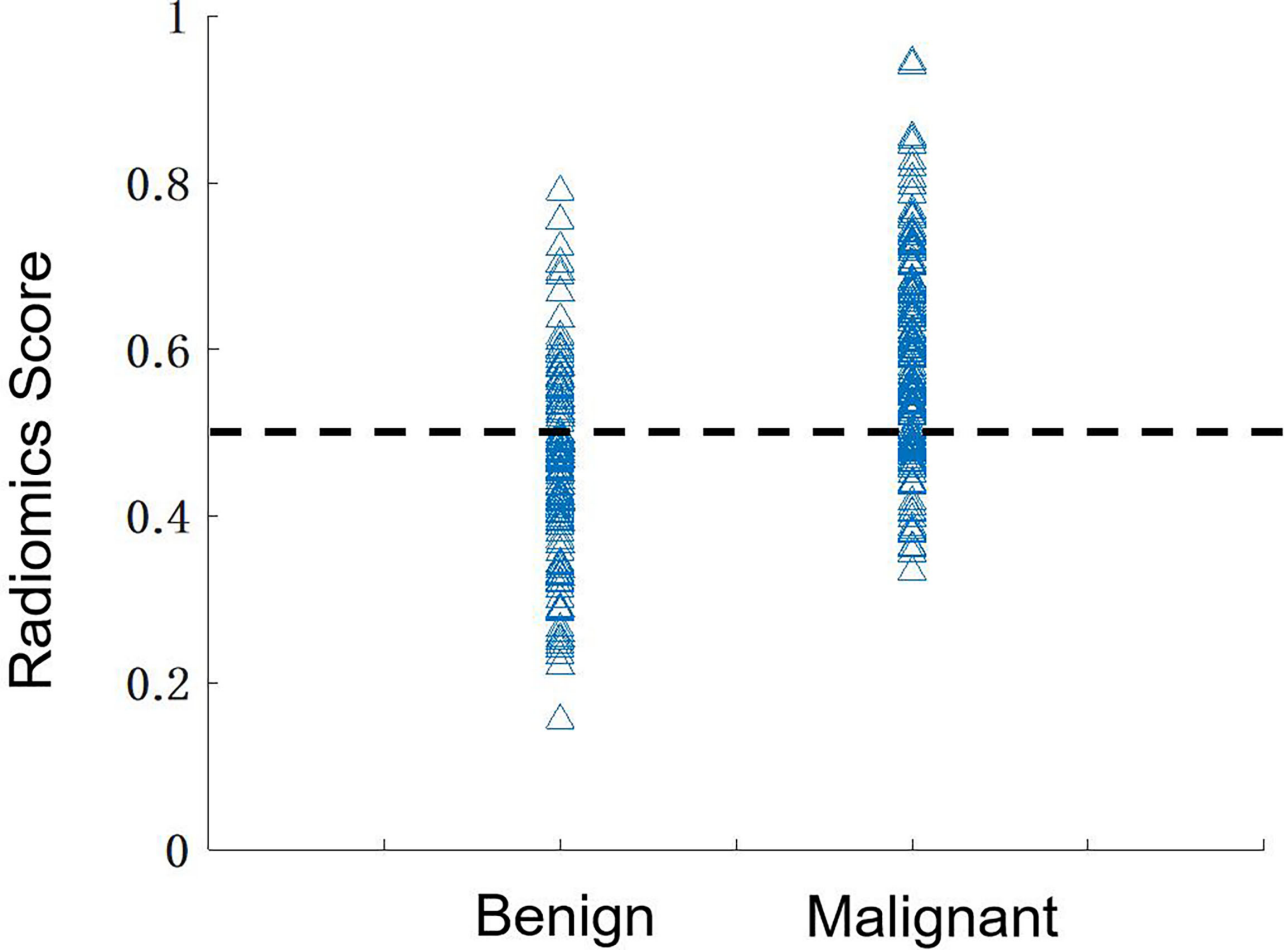
The distribution of the radiomics scores predicted by using the combined model in the benign and malignant groups. By using the threshold of 0.5 as the cut-off value, there are 136 true-positive, 84 true-negative, 39 false-negative, and 39 false-positive cases, with an overall accuracy of 220/298 = 74%.

The diagnostic results are summarized in **Table 3**. The radiomics model built using the combined CC+MLO yielded an AUC of 0.82, sensitivity of 0.78, specificity of 0.68, and accuracy of 0.74. If only features from CC were used, the AUC was 0.77, sensitivity was 0.86, specificity was 0.48, and accuracy was 0.70. If only features from MLO were used, the AUC was 0.72, sensitivity was 0.73, specificity was 0.57, and accuracy was 0.66. The constructed ROC curves are shown in **Figure 4**. From the DeLong’s test, the AUC of the combined CC+MLO model is significantly better than the MLO model (p<0.01). The difference between CC+MLO vs. CC (p=0.10), or CC vs. MLO (p=0.12), did not reach a significant level. **Figure 5** shows the radiomics scores predicted by the combined CC+MLO model in the benign and malignant groups.

**Table 3:** The diagnostic performance of the radiomics models built using CC, MLO, and combined features, and deep learning model built using ResNet50.

Model	Sensitivity	Specificity	Accuracy	AUC
**All Radiomics Features**	0.78 (136/175)	0.68 (84/123)	0.74 (220/298)	0.82
**Radiomics from CC**	0.86 (151/175)	0.48 (59/123)	0.70 (210/298)	0.77
**Radiomics from MLO**	0.73 (127/175)	0.57 (70/123)	0.66 (197/298)	0.72
**ResNet50**	0.53 (93/175)	0.58 (71/123)	0.55 (164/298)	0.61

### Deep learning analysis

**Figure 6:**
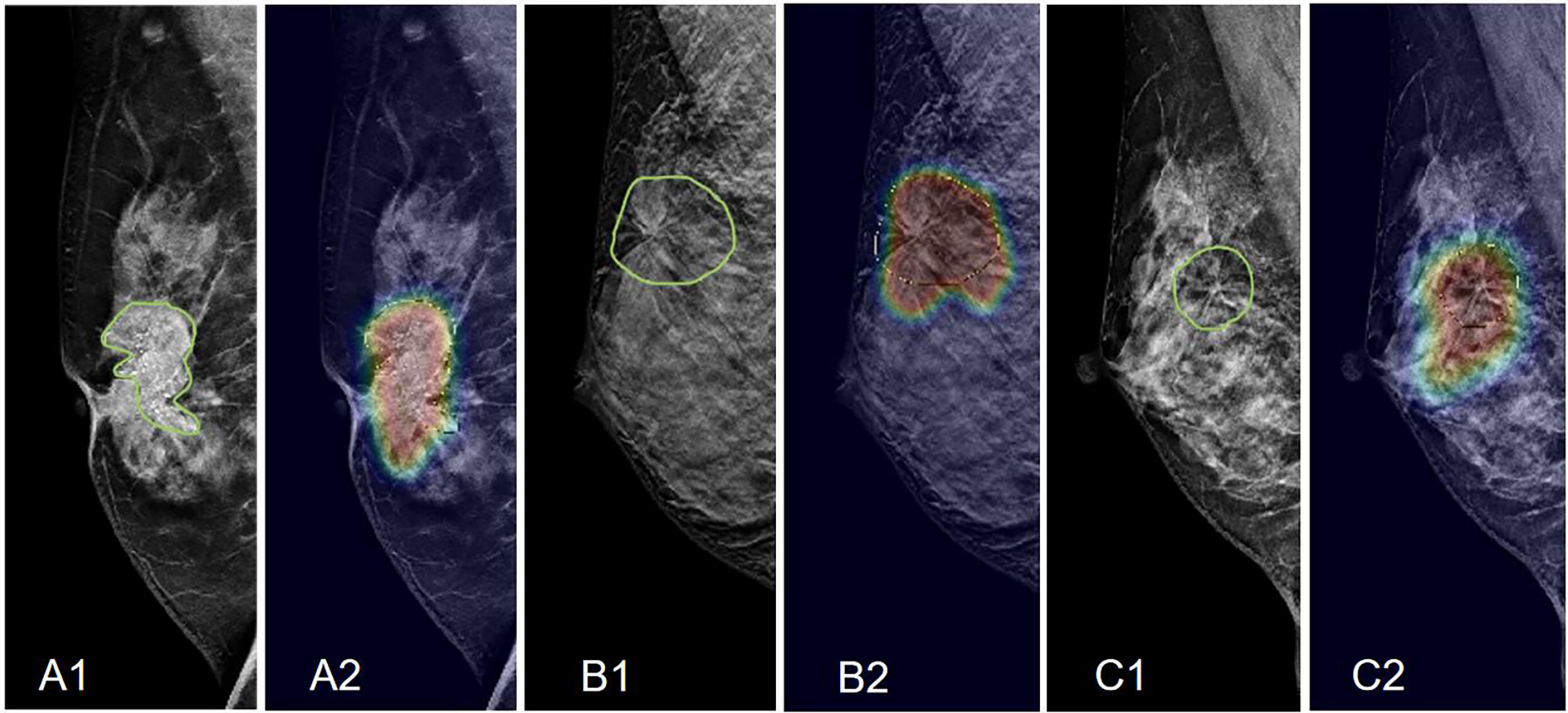
Examples of Grad-CAM maps of architectural distortion on DBT images, predicted by ResNet50 deep learning. **(A)** The RMLO view of a 61-year-old patient diagnosed with invasive ductal cancer. The BI-RADS score is 5. The radiomics score of the combined model is 0.72, and the probability predicted by deep learning is 0.54, both correctly diagnosing this case as malignant. **(B)** The RMLO view of a 42-year-old patient diagnosed with adenosis. The BI-RADS score is 4C. The radiomics score of the combined model is 0.48, and the probability predicted by deep learning is 0.52. The radiomics model makes a correct benign diagnosis, but deep learning gives a false-positive diagnosis. **(C)** The RMLO view of a 46-year-old patient diagnosed with fibroadenoma. The BI-RADS score is 4B. The radiomics score of the combined model is 0.41, and the probability predicted by deep learning is 0.51. The radiomics model makes a correct benign diagnosis, not deep learning. However, although deep learning does not give a correct diagnosis, it can localize the suspicious area.

**Figure 7:**
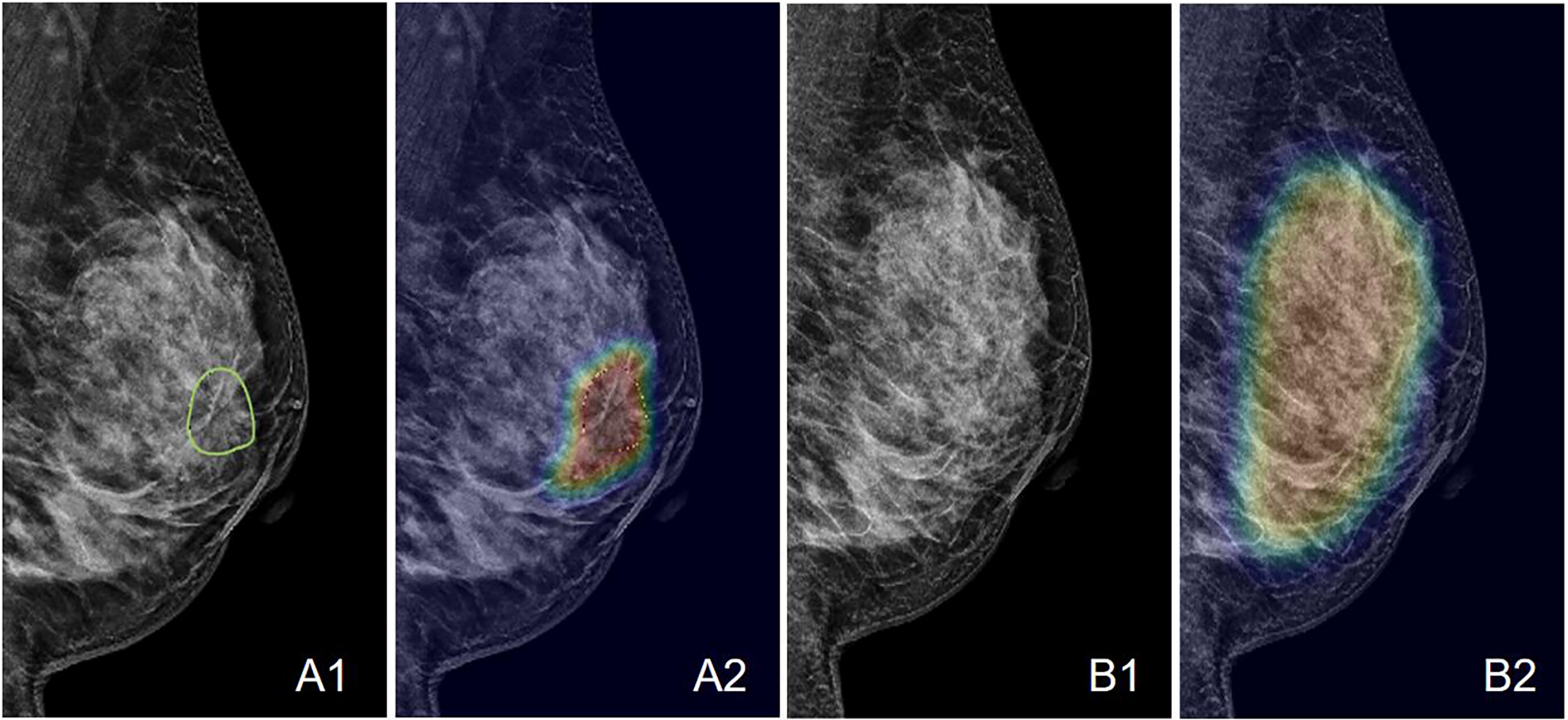
An example of the Grad-CAM map of the architectural distortion in the LMLO view of **(A)** DBT and **(B)** FFDM images of a 53-year-old patient diagnosed with invasive ductal cancer. The BI-RADS score is 4B. The radiomics score is 0.65, and the deep learning probability is 0.62, both correctly diagnosing this case as malignant. When the developed deep learning model from DBT is applied to FFDM, the probability is 0.32, false-negative. The detected suspicious area covers the entire dense tissues, showing the architectural distortion on FFDM cannot be detected.

**Figure 8:**
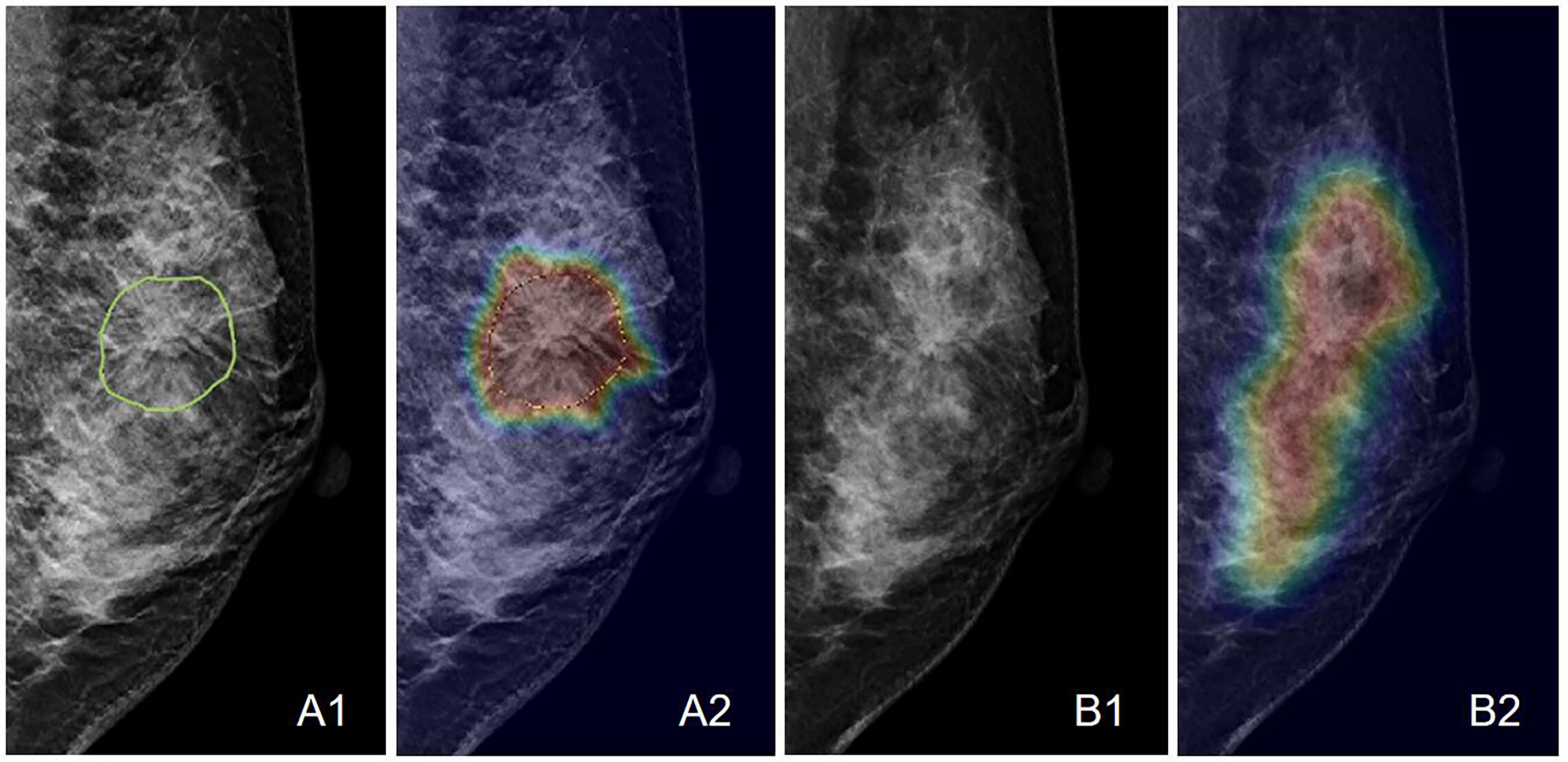
An example of the Grad-CAM map of the architectural distortion in the LMLO view of **(A)** DBT and **(B)** FFDM images of a 46-year-old patient diagnosed with adenosis. The BI-RADS score is 4B. The radiomics score is 0.41, and the deep learning probability is 0.38, both correctly diagnosing this case as benign. When the developed deep learning model from DBT is applied to FFDM, the probability is 0.48, which is also true-negative but reaches the threshold for malignancy. The detected suspicious area covers the entire dense tissues, showing the architectural distortion on FFDM cannot be detected.

The results predicted by the deep-learning model yielded an AUC of 0.61, much worse compared to those achieved by the radiomics models (all significant, p<0.01). This is due to the use of the whole image as input, which is a much more challenging task. One important feature of deep learning is to use the Grad-CAM maps to localize the suspicious area, as shown in **Figures 6** **–** **8**. Although deep learning did not reach a high diagnostic accuracy, it could localize the area with architectural distortion very well. In contrast, when the developed model was applied to the corresponding mammography of the same patient, the detected area was much larger, almost covering the entire dense tissues (**Figures 7**, **8**), and had a worse diagnostic performance. The results suggest that deep learning is highly applicable to analyzing the DBT image to select the suspicious area for further diagnosis, e.g., by using the radiomics models.

## Discussion

In this study, we applied two main AI strategies, including radiomics and deep learning, to diagnose breast cancer in patients presenting with architectural distortion on DBT. This feature has become more noticeable after DBT is extensively applied for breast imaging, as it can better resolve the overlapping tissues compared to the 2D projection mammography. As demonstrated in our dataset, many benign cases also had a high BI-RADS score, 46% ≥ 4B and 83% ≥ 4A. This feature can lead to many false-positive diagnoses and many benign biopsies, and more research is needed to improve the accuracy. In this study, we showed that the radiomics model developed using manually outlined ROI could achieve good accuracy. The AUC of the radiomics model built using features extracted from the combined CC and MLO views was 0.82, which was higher than the AUC of models built using individual views (0.77 for CC, and 0.72 for MLO). In the benign group, 102 of 123 patients (83%) had BI-RADS ≥ 4A, and they would be recommended to receive a biopsy. The specificity of the combined model was 84/123 (68%), and if the biopsy recommendation was made according to the results, only 39 patients would be referred; therefore, the model has the potential to decrease many unnecessary biopsies. The current threshold was based on the probability of 0.5 as malignant, which can be adjusted to a lower value to improve the sensitivity by increasing true positives, but still capable of avoiding many false positives, as shown in Zhou et al. (34).

The deep-learning classification model had a low AUC (0.61), due to the use of the whole image as input. It has been demonstrated that the accuracy of deep learning is highly dependent on the input box size (34). Therefore, the model was trained to predict that there was a malignant lesion somewhere in the image. This is a very challenging task that would normally require a much larger dataset of thousands of images to train. For architectural distortion, it is a much rare feature compared to mass and microcalcifications, and difficult to assemble such a large dataset. Therefore, our main goal is to use deep learning with the Grad-CAM method to detect the architectural distortion areas on DBT images. Then the heat maps can be used to segment the ROI using automatic algorithms for further diagnosis, e.g., by using the developed radiomics models. Grad-CAM is a commonly used method to locate suspicious lesions, and different methods have been reported. Mettivier et al. (32, 33) generated activation maps by using different confident thresholds. We have implemented their methods and found the results generated using both methods were comparable, suggesting the Grad-CAM methods were robust.

We also applied the DBT-trained model with Grad-CAM to the FFDM of the same patients acquired after DBT and showed that architectural distortion was more obvious on DBT than on 2D mammography and that it was difficult to make a diagnosis. In the small set of patients that were tested, the probability generated from the mammography was close to 0.5, which was ambiguous and not able to point to the more likely diagnosis as benign or malignant.

For managing breast cancer, early detection is the cornerstone of preventing morbidity and mortality. Several studies have investigated how architectural distortion detected on DBT should be managed (35–37). According to the BI-RADS lexicon, architectural distortion includes thin straight lines or spiculations radiating from a point, and focal retraction, distortion, or straightening at the anterior or posterior edge of the parenchyma (12). Architectural distortion can also be a secondary finding associated with a primary finding such as a mass or asymmetry (12). The study by Posso et al. found that compared with women who had masses, the highest risk of subsequent breast cancer was found in those with architectural distortions (38). Benign causes of architectural distortion include radial scars, complex sclerosing lesions, sclerosing adenosis, fat necrosis, postprocedural change, and rare spiculated benign lesions, such as breast fibromatosis and granular cell tumor. The major cancer types (IDC and DCIS) can present architectural distortion as a star-shaped pattern. On the other hand, the complex and radial sclerosing lesions presenting with architectural distortion with larger than 1 cm are probably benign (11). Studies have also shown that invasive lobular carcinomas (ILC) are highly associated with architectural distortions (39, 40).

All the patients in our study had confirmed pathological results, with 175/298 (59%) malignant, and 123/289 (41%) benign. There is a high chance of malignancy, and it is necessary to pay attention to the architectural distortions detected by DBT (41). The results are consistent with those reported by Pujara et al. and Ambinder et al. (37, 42). DBT reduces the superimposition of fibroglandular tissues, thereby improving visualization of findings that may be subtle or occult on DM, particularly the architectural distortion (37, 43, 44). Ahmed et al. showed that DBT-detected architectural distortion is less likely to represent malignancy compared to those detected on DM; however, the risk of malignancy is not low enough to forgo biopsy (45). DBT-guided biopsy has been demonstrated to be feasible, safe, and effective for the pathologic diagnosis of lesions presenting with architectural distortion and may be particularly valuable for the detection of early-stage malignancies (41, 46). Nevertheless, considering the risks of procedures and the psychological burden on the patients, the best approach for the low-risk lesions may be imaging surveillance rather than biopsy/surgery. In a recent study by Villa-Camacho et al., the upgrade rates of architectural distortion on DBT from nonmalignant pathology at biopsy to malignancy at surgery were investigated (35). It was reported that nonmalignant pathology at biopsy has an overall upgrade rate to malignancy at the surgery of 10.2%, but architectural distortion without atypia has a low upgrade rate of 2.2% (35).

Architectural distortion is a particularly challenging pattern for radiologists as it may be difficult to discern from the normal overlapping of the various soft tissue, parenchyma, vessels, and density ligamentous structures (47). In fact, due to its subtle nature, architectural distortion has been shown to have poor interobserver reproducibility in terms of agreement for recall among radiologists compared with masses and calcifications (43).

Furthermore, not all architectural distortions appear like thin straight lines or spiculation radiating from a point. Some atypical features are difficult to detect due to the lack of common characteristics, as shown in the case examples in **Figure 3**. Radiologists need long-term training to detect these atypical architectural distortions. In our study, we used Grad-CAM to localize the distortion areas by generating gradient heat maps. The results suggest that deep learning can provide a tool to aid in the localization of the distortions in the images. It has the potential to reduce the intra- or inter-reader variation.

Li et al. proposed a deep-learning-based model that used mammary gland distribution as prior information to detect architectural distortions in DBT (48). The proposed network was faster-RCNN, which has been proven capable of yielding a satisfactory performance to search and detect lesions in medical images. However, due to the difficulty to obtain the ground truth of the distortion on DBT, the training was difficult, and further hampered by the limited cases because architectural distortion is not a common feature. In our study, we did not train a detection-specified network, but used Grad-CAM to visualize the suspicious areas, which can help physicians, especially inexperienced junior physicians, to detect and diagnose architectural distortion or other unclear abnormalities.

There are some limitations in our study. First of all, the ROI was delineated manually. As shown in the case examples, the architectural distortion was a subtle feature, and it did not have a clear boundary that could be traced, so the drawing was done by encompassing all abnormal areas. It was not practical to compare the ROI drawing done by different readers, so we used the consensus, verified by an experienced radiologist. Secondly, only the most obvious distortion shown on one DBT slice was used in the analysis. The performance by using the ROI’s from multiple slices needs to be investigated. One advantage of DBT compared to 2D mammography was that the distortion can be seen clearly on one slice, so we started with a single-slice approach. Thirdly, for model training, particularly using deep learning, a much larger dataset is required. The developed models will need to be tested using an independent dataset to validate the performance. Nonetheless, the present study should be able to lay down a good foundation for future studies. After the models are validated, such a tool may assist radiologists in diagnosing architectural distortion, especially for junior and inexperienced radiologists. If a case has a very high benign possibility, a follow-up recommendation (3, 6 months, or even one year) can be given to avoid biopsy or surgery.

## Conclusion

In this study, we demonstrated that for the diagnosis of architectural distortion detected on DBT, the radiomics model can achieve satisfactory diagnostic accuracy. Although the accuracy of deep learning was low, the trained model could enable the Grad-CAM to localize the suspicious areas showing architectural distortion, which could be used for automatic ROI delineation. Our study may provide a helpful computer-aided diagnostic tool for first detecting subtle pathological textures on DBT images, and then for further characterization to make a diagnosis. The radiomics analysis is a commonly applied, mature, method for computer-aided diagnosis. As shown in our study, it has the potential to improve the specificity of the DBT-detected architectural distortion and reduce unnecessary biopsies and surgeries, while maintaining a high sensitivity for the diagnosis of breast cancer.

## Data availability statement

The original contributions presented in the study are included in the article/supplementary material. Further inquiries can be directed to the corresponding authors.

## Ethics statement

The studies involving human participants were reviewed and approved by ethics committee in Clinical Research (ECCR) of the First Affiliated Hospital of Wenzhou Medical University (No.2020063). Written informed consent for participation was not required for this study in accordance with the national legislation and the institutional requirements.

## Author contributions

XC, YZ, and M-YS conceptualized and designed the study. GC and MW provided administrative support. XC, XiaL, and JZ provided the study materials or patients. XC, WH and JZ collected and assembled the data. XC, YZ, XW, KN, and M-YS analyzed and interpreted the data. XC, YZ, XW, XiaL, XinL, and M-YS wrote the manuscript. XC, YZ, XW, KN, XiaL, XinL, JZ, WH, M-YS, MW, and GC gave the final approval of the manuscript. All authors contributed to the article and approved the submitted version.
